# Revisiting Pleiotropic Effects of Type I Interferons: Rationale for Its Prophylactic and Therapeutic Use Against SARS-CoV-2

**DOI:** 10.3389/fimmu.2021.655528

**Published:** 2021-03-26

**Authors:** Diana Garcia-del-Barco, Daniela Risco-Acevedo, Jorge Berlanga-Acosta, Frank Daniel Martos-Benítez, Gerardo Guillén-Nieto

**Affiliations:** ^1^Neuroprotection Project, Center for Genetic Engineering and Biotechnology, Pharmaceutical Division, Havana, Cuba; ^2^Cytoprotection Project, Center for Genetic Engineering and Biotechnology, Pharmaceutical Division, Havana, Cuba; ^3^Intensive Care Unit 8B, Hermanos Ameijeiras Hospital, Havana, Cuba; ^4^Biomedical Research Direction, Center for Genetic Engineering and Biotechnology, Havana, Cuba

**Keywords:** type I interferons, SARS-CoV-2, COVID-19, ACE2, neutrophil-mediated inflammation

## Abstract

The pandemic distribution of SARS-CoV-2 together with its particular feature of inactivating the interferon-based endogenous response and accordingly, impairing the innate immunity, has become a challenge for the international scientific and medical community. Fortunately, recombinant interferons as therapeutic products have accumulated a long history of beneficial therapeutic results in the treatment of chronic and acute viral diseases and also in the therapy of some types of cancer. One of the first antiviral treatments during the onset of COVID-19 in China was based on the use of recombinant interferon alfa 2b, so many clinicians began to use it, not only as therapy but also as a prophylactic approach, mainly in medical personnel. At the same time, basic research on interferons provided new insights that have contributed to a much better understanding of how treatment with interferons, initially considered as antivirals, actually has a much broader pharmacological scope. In this review, we briefly describe interferons, how they are induced in the event of a viral infection, and how they elicit signaling after contact with their specific receptor on target cells. Additionally, some of the genes stimulated by type I interferons are described, as well as the way interferon-mediated signaling is torpedoed by coronaviruses and in particular by SARS-CoV-2. Angiotensin converting enzyme 2 (ACE2) gene is one of the interferon response genes. Although for many scientists this fact could result in an adverse effect of interferon treatment in COVID-19 patients, ACE2 expression contributes to the balance of the renin-angiotensin system, which is greatly affected by SARS-CoV-2 in its internalization into the cell. This manuscript also includes the relationship between type I interferons and neutrophils, NETosis, and interleukin 17. Finally, under the subtitle of “take-home messages”, we discuss the rationale behind a timely treatment with interferons in the context of COVID-19 is emphasized.

## Introduction

Severe acute respiratory syndrome (SARS) is an infectious disease of this century caused by coronaviruses (SARS-CoV and SARS-CoV-2) that leads to pulmonary and other systemic pathological conditions (1–3). Viruses deploy different strategies to circumvent the antiviral actions of the innate immune response. SARS-CoV-2, as well as its related coronaviruses SARS-CoV and MERS, is a virus that encodes an array of proteins able to impair type I and III interferon signaling and the subsequent activation of innate immune response (4–6).

Besides the mandatory hospitalization of coronavirus diseases 2019 (COVID-19) positive patients and the isolation of their epidemiological chain contacts, the entire Cuban therapeutic approach has been crucial. Among the strengths of the latter, the early administration of interferon alpha 2b (IFNα2b) (Heberon Alfa R, Cuba) could has contributed to the effective control of COVID-19 in Cuba reducing the high incidence of severe cases and mortality (see covid19cubadata.github.io/#cuba). This IFNα2b-based treatment was used not only on patients suffering COVID-19 symptoms, but also on their asymptomatic confirmed and suspected epidemiological contacts, as well as on health professionals at risk (7).

During a viral infection, the most prominent cytokines produced are interferons (IFNs) (8), which represent the major effector cytokines of the host immune response against viruses (9). Traditionally, a dual role is attributed to type I IFN: directly inhibiting viral replication and eliciting an immune response to clear virus infection (10, 11). However, present knowledge reveals that the scope of type I IFN is much broader. In this article we have attempted to review the potential therapeutic horizons of the IFN alpha (IFN-α) system against SARS-CoV-2 virus. The present review is intended to analyze the contribution of IFN-α therapies in the context of SARS-CoV-2 on the rationale of molecular biology, genetics, and the immune response elicited by IFNα2b.

To accomplish this goal we have reviewed literature indexed in PUBMED from 1980-2020, restricted to English language. Articles available in Cuban and international repositories were also considered. We expect that this review will help scientists and clinicians on the Covid-19 battlefield to understand and systematize their knowledge about interferons.

## Type I IFN

Type I IFNs have a pivotal role inducing an antiviral state (12) in non-immune cells while orchestrating antiviral immune responses through several mechanisms. These mechanisms include the inhibition of viral replication in infected cells, potentiating antigen presentation and sustaining the adaptive immune response by a direct and indirect effect on T and B cells that constitute the immunological memory response (13, 14).

IFNs are small protein and glycoprotein cytokines produced by leucocytes, T-lymphocytes, and fibroblasts in response to infections and other biological stimuli after recognition of pathogenic components mediated by pattern recognition receptors (PRRs) (15). Although most mammalian nucleated cells are capable of producing type I IFNs (12), plasmacytoid dendritic cells (pDCs) are the professional IFN producer cells (16). IFNs do not only function as direct antiviral proteins, they also have several other biological properties such as inhibition of cellular proliferation, immunomodulation (4) and even desensitization after activation of immune response (13, 17), making their role in viral infections broader than just their direct antiviral activity.

IFN-α has 13 subtypes and, along with IFN-β, IFN-ϵ, IFN-κ, and IFN-ω, it belongs to the type I IFNs, which is the largest IFN class (4, 11). The genes for the different type I IFNs are all located together, on chromosome nine (18).

All IFNs initiate their biological effects by binding to specific receptors expressed on the cell surface. Upon induction, type I IFNs act in an autocrine, paracrine, or systemic manner to stimulate a range of responses. The best-characterized function is the ability of type I IFNs to induce an antiviral state into the cell through upregulation of antiviral genes (12, 19, 20). IFN signaling is context-specific (12), thus in virally infected cells type I IFN signaling enhances the susceptibility to undergo apoptosis, thereby, preventing viral replication and spread (12, 21). Dendritic cells (DC) response to type I IFN consists of their activation and secretion of proinflammatory cytokines that lead to activation of the adaptive immune response (22). After exposure to type I IFN Natural Killer (NK) cells exacerbate their potent killer ability targeting virally infected cells (23, 24) and pDCs, which secrete extremely high levels of type I IFNs, promote B cells activation and the subsequent production of antiviral antibodies (16, 25).

## Induction of Type I INFs in Response to Viral Infections

**Figure 1:**
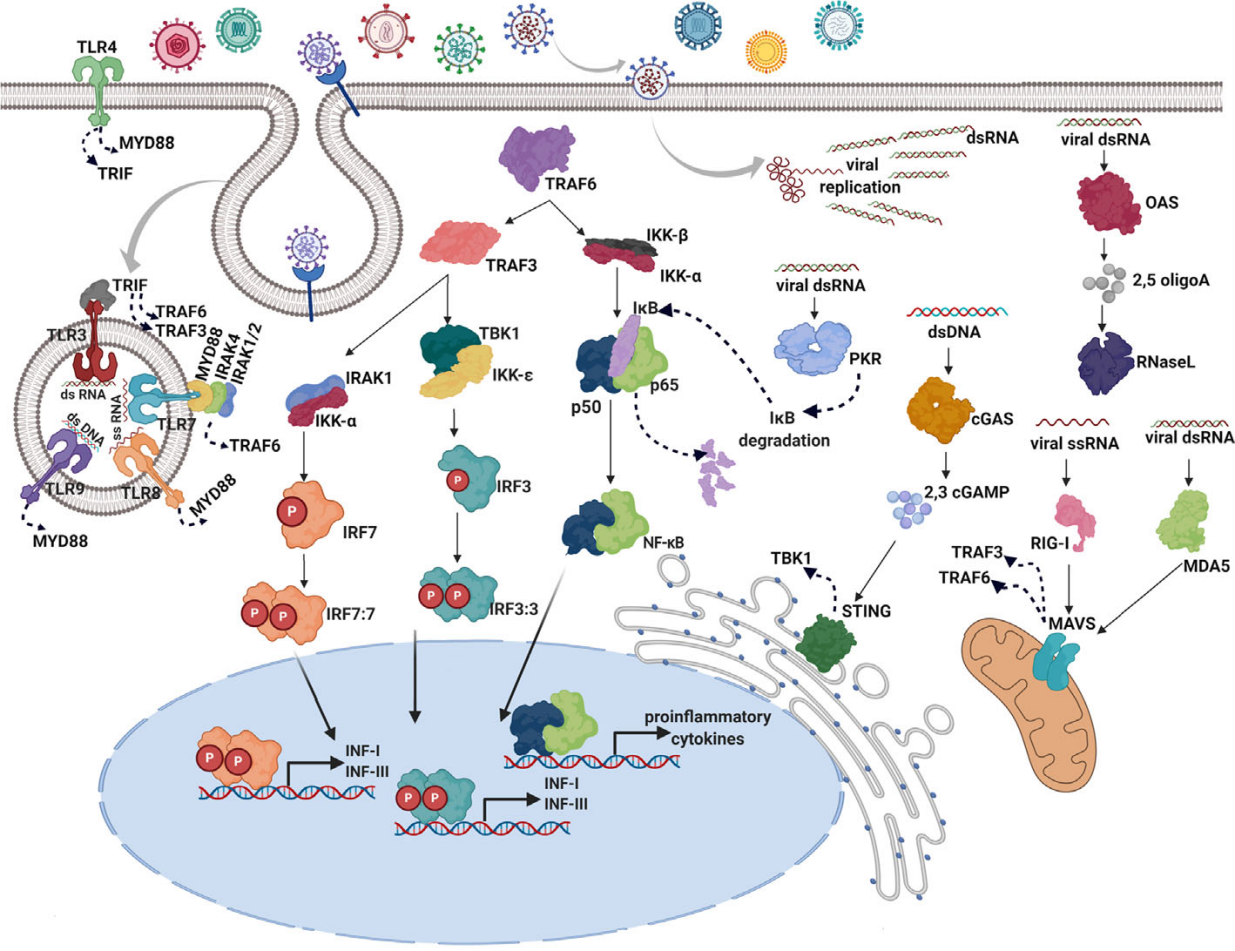
Induction of type I IFNs in response to viral infections. After virus entry through cell membrane using receptors or membrane fusion strategies, the infected cell detects the presence of virus replication by PRRs. There are two IFN-inducing systems in response to viral PRRs: one system is ubiquitously expressed and localized in the cytoplasm (OAS, MDA-5, RIG-I, cGAS, and PKR), wherein viral nucleic acids produced upon infection are detected, the other one is restricted to endosomes of specialized sentinel cells and mediated by Toll-like receptor (TLR) family (TLR3,7,8,9) (12). In the cytosol, viral double-stranded (ds) RNA is recognized by melanoma differentiation-associated protein 5 (MDA-5), 2’-5’-oligoadenylate synthetase (OAS), and protein kinase R (PKR) whereas retinoic acid-inducible gene I (RIG-I) is specialized in single-stranded (ss) RNA detection. Cyclic GMP-AMP synthase (cGAS) acts as a double-stranded DNA sensor (27). In the endosomes, TLRs are transmembrane proteins that detect double-stranded RNA (dsRNA; TLR3), single-stranded RNA (ssRNA; TLR7 and TLR8), and double-stranded DNA (dsDNA; TLR9). Additionally, TLR4 is located on the cell surface, to detects viral glycoproteins (28). Activation of RIG-I and MAD-5 leads to activation of mitochondrial antiviral-signaling protein (MAVS) in the mitochondrion and the consequent signaling through TNF receptor associated factors (TRAFs) such as TRAF3 and TRAF6 (29). Detection of viral material by OAS leads to the synthesis of 2’-5’-oligoadenylates, which then act as intracellular second messengers to activate latent endoribonuclease (RNase L) reinforcing the innate response through indiscriminate cleavage of host and viral RNA and producing additional PAMPS (30). In response to the activation of the cGAS nucleotidyltransferase activity, cyclic GMP-AMP (cGAMP) is produced, which in turn binds to and activates the stimulator of IFN genes (STING) in the endoplasmic reticulum, inducing the TANK-binding kinase 1 (TBK1) activation (31). PKR activation leads to a dramatic reduction of host and viral translation and degradation of κB inhibitor (IκB), resulting in the activation of the NF-κB signaling pathway (32). Upon TLR viral recognition, activation of adapters, myeloid differentiation primary response 88 (MYD88) and TIR-domain-containing adapter-inducing interferon-β (TRIF) occurs, leading to the activation of TRAF6 and TRAF3 (33). Immediately upstream of the IRFs, IκB kinase‑ϵ (IKKϵ) and TANK-binding kinase 1 (TBK1) are responsible for the phosphorylation of IRF3 and IRF7 (34). Once activated IRF3, IRF7, and NF-κB are translocated to the nucleus where they bind to specific binding sites, turning on the transcription of type I and III IFN genes and proinflammatory cytokines (6).

Most nucleated cells respond against viral infection by producing type I IFNs (12), which represent the first line of defense against many diverse pathogens (10). Type I IFNs are induced after pathogenic infection *via* detection of pathogen-associated molecular patterns (PAMPs) and damage/danger-associated molecular patterns (DAMPs) by innate PRRs (26). The induced signaling cascades activate IFN-regulatory factors (IRF) 3, IRF7, and nuclear factor kappa-light-chain-enhancer of activated B cells (NF-kB), resulting in the production of type I IFNs and pro-inflammatory cytokines (**Figure 1**).

**Figure 1** illustrates the complex, entangled, and redundant induction of type I IFN by viral cues.

## Type I IFN Signaling

**Figure 2:**
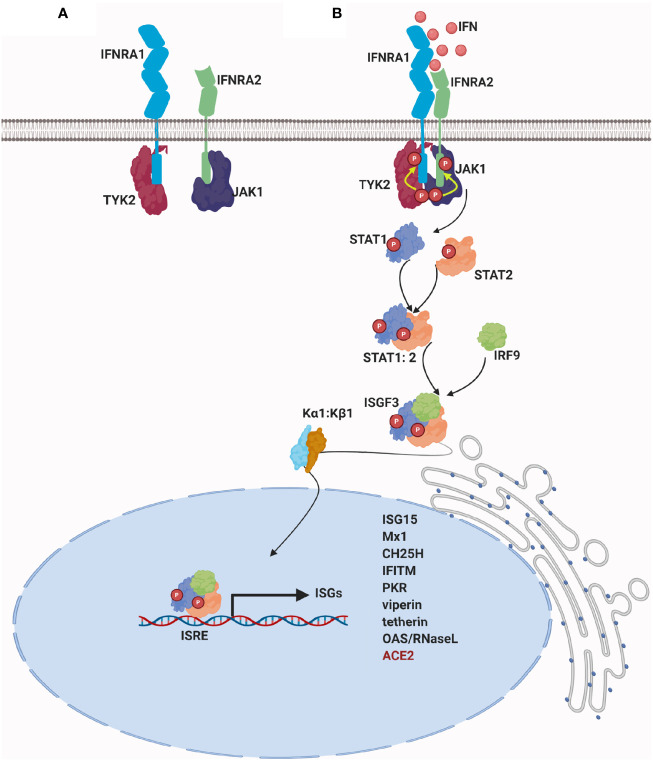
Signaling of type I IFN: **(A)** In the absence of a stimulus, a specific JAK protein in an inactive conformation binds constitutively to the cytoplasmic domain of each IFN receptor chain: IFNAR1 associates with tyrosine kinase 2 (Tyk2) and IFNAR2 associates with tyrosine kinase Janus 1(JAK1) (36). **(B)** Upon IFN binding, receptor chains are brought into close proximity, and the two JAK kinase domains remain juxtaposed and undergo transphosphorylation and sustained activation. Once activated, JAKs phosphorylate IFN receptor´s chains on highly conserved tyrosine residues, which lead to the repositioning or binding of STAT proteins (37). As a result, STATs are phosphorylated on conserved tyrosine residues and they are dissociated from the receptor (21). Structural changes of activated STATs leads to heterodimerization of STAT1 and STAT2 and to its interaction with IFN regulatory factor 9 (IRF9). Binding of STAT1, STAT2, and IRF9 results in the formation of a complex called IFN-stimulated gene factor 3 (ISGF3) (38–40). ISGF3 complex is normally translocated into the nucleus by binding to karyopherin alpha 1 (KPNA1) and recruits karyopherin beta 1(KPNB1) (41). ISGF3 binds IFN-stimulated regulatory elements (ISREs) in the DNA upstream of IFN-stimulated genes (ISGs), resulting in the transcription of hundreds of type I ISGs. For further details visit (10, 11, 19, 21, 42).

Type I IFNs bind to the transmembrane type I IFN receptors (IFNAR), which have a multisubunit structure; IFNAR1 and IFNAR2 subunits (35). Typically, conformational changes in the intracellular portion of the receptor lead to the activation of the Janus kinase (JAK) family and the activation of signal transducer and activator of transcription (STAT) signaling pathway (4). STAT proteins form heterodimers and are translocated to the nucleus where they bind to specific sequences, thus regulating gene transcription of hundreds of genes (21) (**Figure 2**).

## IFN-Stimulated Genes

Although the regulation of the IFN-stimulated genes (ISGs) is beyond the scope of this review, here we provide a brief illustration of its action mechanism.

Many of the proteins encoded by these ISGs, cope together to reach certain cellular outcomes, such as cell-intrinsic antiviral defense, antiproliferative activities, and stimulation of adaptive immunity (42) or other processes such as inflammation, and the impairment of viral entry, replication, and egress (11, 43, 44). The ISGs act by several mechanisms such as a slowdown of cell metabolism or the activation of adaptive immunity mediated by cytokines. Exemplary ISGs include PRRs which sense aberrant RNA structures formed during virus replication (45). Other ISGs associated with SARS-CoV-2 pathogenicity are Cholesterol-25-hydroxylase (CH25H) which converts cholesterol to a soluble antiviral factor and is a potent SARS-CoV-2 inhibitor (46), and the type I IFN-inducible transmembrane protein family (IFITM), that during SARS-CoV-2 infection may paradoxically act as an entry cofactor in human lung cells (47). One of the most highly upregulated ISGs is a 15-kDa ubiquitin-like protein, (ISG15), which covalently links to viral and host target proteins altering the ability to engage in their typical interactions (48), thereby regulating protein stability, traffic, and function, finally inhibiting viral replication (49). ISGylation is the process of ISG15 to covalently link to certain proteins. Papain-like protease (PLpro) encoded by SARS-CoV-2 is responsible for the suppression of host innate immune responses through the reversal post-translational modification of proteins performed by ISG15 (50). Several proteins involved in ISGylation are also induced by IFNs, for example, ISGylation of IRF3 prevents PIN1 binding, a protein that promotes IRF3 ubiquitination, thus increasing IRF3 stability and sustaining its activity as a transcription factor (51).

Being IFN expression an accurately controlled process, after IFN exposure the cells undergo an IFN-desensitized state which allows them to recover from IFN signaling and thus avoid an exacerbated immunological activation that may result in tissue damage and organ failure, as occurs during uncontrolled inflammatory responses to viral infection associated to cytokine storm and high mortality (13, 17). Some IFN desensitization mechanisms are mediated by ISGs (11). During SARS, the dysregulated type I IFN responses may end up in a disrupted switch from hyper-immune to protective adaptive immune responses in the host, prevailing a more severe condition afforded by the unremitting induction of inflammatory cytokines (52). This is just the tip of the iceberg concerning to the magnitude and diversity of ISGs impact on IFNs physiology.

## IFNs Signaling Targeted by Coronavirus

Unlike pathogens as bacteria and fungi, viruses are made of host-derived components, thus they lack highly conserved invariant structures that could alert the immune system. Typically, cells recognize viral infection by viral nucleic acids. Receptors capable of detecting viruses are highly successful in differentiating host-derived RNA and DNA from viral nucleic acids, and this is also supported by the compartmentalization of antiviral sensors (53). Type I IFN cytokine family is intended to signal the presence of intracellular infection and enables communication among the cells that provide defense against viruses or intracellular bacteria (12). Viruses are known to target both type I IFN receptor signaling as well as the signaling machinery that links cytosolic nucleic acid recognition to activation of type I IFNs (23, 24). SARS-CoV-2 is a poor inducer of type I IFN response (6) which has been also confirmed by reduced type I IFN levels in the serum of SARS-CoV-2 infected patients, which is associated with a poor outcome (5, 54).

**Figure 3:**
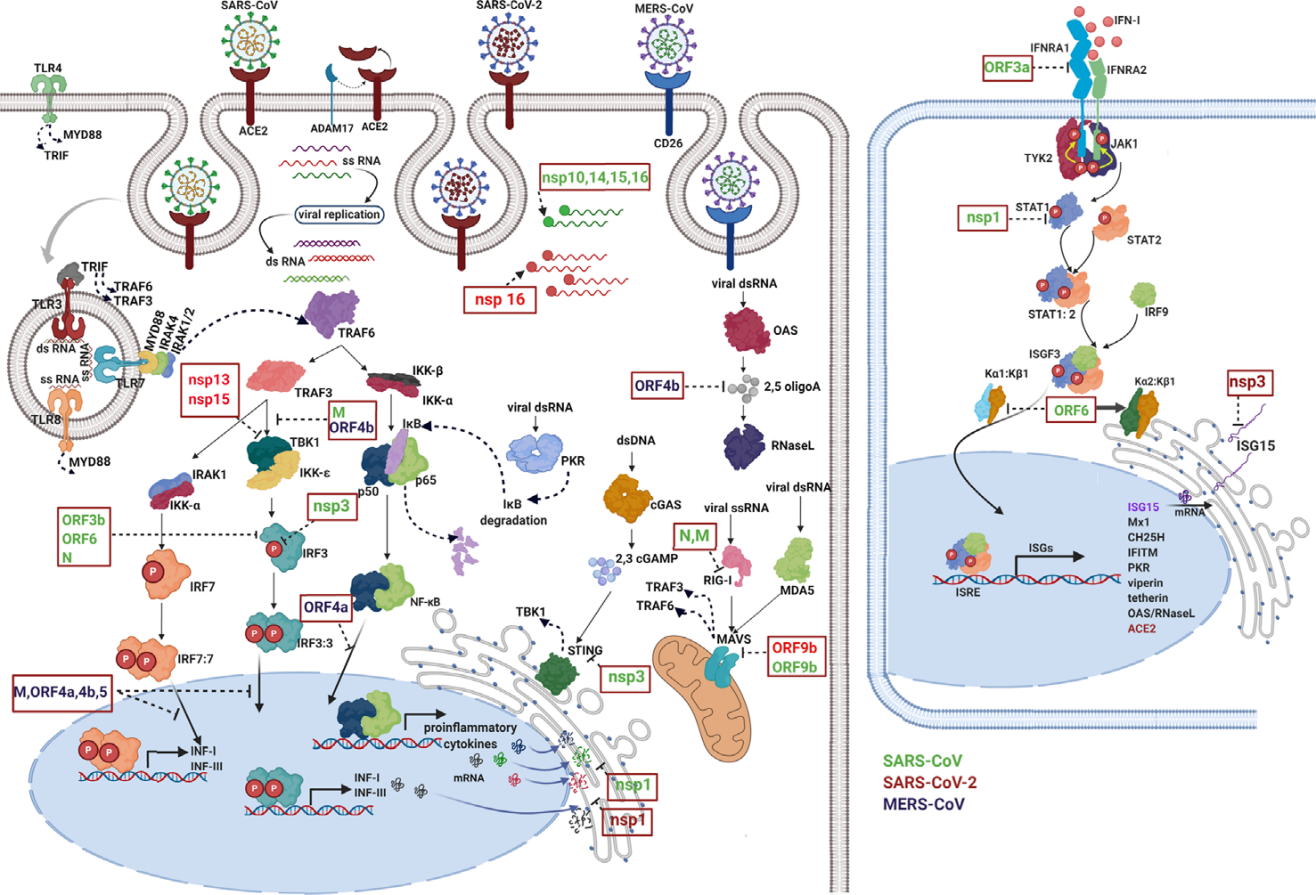
Immune evasion by coronaviruses. The image schematically represents how viral proteins of Betacoronavirus (green: SARS-CoV, red: SARS-CoV-2, and purple MERS-CoV) are capable of inhibit different events of the immune response such as pathogen detection, IFN production, IFN signaling, and ISG functions. Each viral protein blocks one or more key signaling proteins. First, viruses modify their nucleic acids to avoid being recognized by cytosolic receptors. Viral RNA can be guanosine-capped and methylated at the 5’ end by SARS-CoV nonstructural proteins (nsp 10, nsp14, nsp15, and nsp16), allowing the virus to efficiently evade recognition of host dsRNA sensors (57–62). Viral proteins inactivate key intermediates in the recognition of pathogens, for example, RIG-I activation is blocked by SARS-CoV N protein (63) and M protein the latter is physically associated with RIG-I (64). Additionally, other viral proteins inhibit different intermediate steps in the signaling cascade for the production of IFN, for example: ORF9b from SARS-CoV-2 and SARS-CoV indirectly interact with MAVS in mitochondria (65, 66). Signaling through STING in the endoplasmic reticulum is antagonized by SARS-CoV nsp3 (67). Viral proteins interfere with complex formation such as TBK-1, which is inhibited by SARS-CoV M and MERS-CoV ORF4b (64, 68). Also, SARS-CoV-2 nsp13 and nsp15 interfere with TBK-1 signaling and IRF3 activation (66). Phosphorylation reactions and translocation to the nucleus of key proteins in the signaling cascade are also targeted by viral proteins, for example, SARS-CoV nsp3, N, ORF3b and ORF6 (69, 70). Viral proteins are even capable of inhibiting the function of transcriptional factors specific for the induction of INFs and proinflammatory cytokines. Proteins of MERS such as ORF 4a, ORF 4b, ORF 5, and M inhibit IRF-3/IRF-7 function and ORF 4a inhibits NF-κB function (68). Additionally, nsp1 protein of SARS-CoV and SARS-CoV-2 suppresses host gene expression by promoting mRNA degradation leading to a strong protein synthesis inhibition, including proteins involved in host innate immune functions (71, 72). The right panel shows how several viral proteins block IFN signaling, SARS-CoV ORF3a protein induces degradation of IFN alpha-receptor subunit 1 (IFNAR1) (73). SARS-CoV nsp1 significantly inhibited IFN-dependent signaling by decreasing the phosphorylation levels of STAT1 (74). Additionally, SARS-CoV ORF6 protein inhibits nuclear translocation but not phosphorylation of STAT1, this occurs through the recruitment of karyopherin (α2 and β1) in the endoplasmic reticulum, thus preventing the translocation of the ISGF-3 complex to the nucleus (75). Finally, coronaviruses inhibit the effector function of ISGs, for example, MERS-COV ORF4b antagonizes activation of the OAS-RNase L pathway through its 2,5–phosphodiesterase activity that degrades the products of OAS (2,5 oligoA) which prevent activation of RNase L (76). Nsp3 encoded by SARS-CoV-2 is responsible for the suppression of host innate immune responses through the reversal post-translational modification of proteins performed by ISG15 (50). For further details visit (6).

Interactions between the adaptive immune system and coronavirus have been deeply studied. SARS-CoV and MERS-CoV are coronaviruses closely linked with SARS-CoV-2: phylogenetic analysis reveals about 79% and 50% similarity, respectively (55). Despite differences in their epidemiology, pathology, and in several of their proteins, the three coronavirus types have similar properties (56) and display several mechanisms for inhibiting the induction of type I IFNs (**Figure 3**).

Solid shreds of evidence show that the host response to SARS-CoV-2 fails to orchestrate a robust IFN response while simultaneously inducing high levels of chemokines supporting improper recruitment of effector cells (6). In such a case, exogenous IFN delivery may succeed to balance the relative scarcity of IFN-induced antiviral effects in the context of exacerbated recruitment of immune effector cells. Moreover, *in vitro* experiments have demonstrated that type I IFN inhibits replication of SARS-CoV-2 in infected cells (6, 77, 78), hence therapeutic delivery of IFNs could avoid SARS-CoV-2 replication.

## Type I IFNs: Preclinical and Clinical Studies

IFN-α has been claimed efficient in treating coronavirus-induced respiratory diseases (79, 80). Notwithstanding IFNs should be administered during early stages of viremia since the stimulated inflammatory cytokines help to control the viral load. When IFN administration overlaps with the already established cytokine storm induced by high viremia, the effect of exogenous IFN is instead deleterious: late IFN intervention elicits exacerbated cytokine production (81), and impairs lung epithelial regeneration (82). Thus, the timing of the IFN intervention is crucial to avoid adverse events.

Type I IFN therapeutic approaches have been studied against MERS-CoV and SARS-CoV both *in vitro* and *in vivo* (83). Both coronaviruses are able to disrupt the IFN signaling pathway (84). Recent articles report that IFN pathways are also disrupted by SARS-CoV-2 (5).

As MERS-CoV and SARS-CoV coronaviruses are closely linked to SARS-CoV-2, the knowledge provided from experiments using type I IFN treatment against these agents may be transposed into the clinical arena of SARS-CoV-2 as a potential treatment. An advantageous feature of SARS-CoV-2 in the context of IFN-α therapy is its increased sensitivity to IFN over SARS-CoV, since the former induces STAT1 phosphorylation and ISGs expression, which is absent in the SARS-CoV action mechanism (77). Additionally, the loss of SARS-CoV-2 ORF6 anti-IFN function (84) renders the virus much more susceptible to type I IFN treatment because activated STAT1 enters the nucleus, induces ISGs and elicits the subsequent antiviral response (77).

Preclinical studies showed that pegylated IFN-α mediates the protection of type 1 pneumocytes against SARS-CoV infection in macaques (85). Moreover, type I IFN administration shortly after MERS-CoV challenges in mice, protected mice from lethal infection even in a scenario of decreased ISGs and inflammatory cytokine gene expression, contrasting with failure and side-effects elicited by delayed IFN delivery (86).

During SARS, MERS, and more recently SARS-CoV-2 outbreaks, IFNs have been generally used in combination with other antiviral drugs (87–94). This combined approach has shadowed the plethora of antiviral effects of exogenous IFN. Indeed, the impact of IFN-α itself might be enough to control the viral load at the beginning of the viral infection and thus preclude the subsequent severe course of COVID-19 disease, while resembling a physiological endogenous response to viral infections and avoiding side effects of synthetic antivirals (95, 96).

At the beginning of COVID-19 there were few studies concerning type I IFN as a standalone treatment. In one of them, IFNα2b delivered by aerosol (using nebulizer and mask), accelerated viral clearance compared to arbidol treatment alone (97). Another study conducted by Sheng and Yang showed that IFNα2b, used as a nasal spray, reduced the infection rate of the respiratory syncytial virus, influenza virus, adenovirus, and SARS-CoV (98). Recently, an investigator-initiated open-label study showed that recombinant human IFN-α nasal drops may effectively prevent COVID-19 in medical staff, as a prophylactic approach together with the standard physical isolation (99). The report by Wang et al. has a core significance as it demonstrates that early onset of type I IFN therapy is associated with reduced mortality and a better response as compared to classical antivirals (100).

One year after SARS-CoV-2 became pandemic, there are several clinical trials evaluating interferons both as the main drug intervention and as a component of the standard therapy (see web sites https://www.clinicaltrials.gov/ and https://rpcec.sld.cu/ from ClinicalTrials.gov and Cuban Public Registry of Clinical Trials, respectively). So far, (February 2021) there are 42 registered trials in which interferons are the main subject matter. Those trials are based on different interferon types (alpha, beta, lambda and gamma), on groups of tributary patients, on therapeutic or prophylactic approaches, on delivery routes, and so forth (**Table 1**). Although most of those studies are not yet completed, the increased use of interferons for the treatment of COVID-19 patients highlights its significance among the scientific and medical community. However, almost none of the abovementioned trials included as endpoints parameters associated to RAS (oxygen saturation and blood pressure), neutrophil-lymphocyte relationship, regulatory T cells elicitation, interleukin 17 and Th17 cells inhibition all of which are thoroughly analyzed in this review.

**Table 1:** Current clinical trials based on Interferons for COVID-19.

IFN type/Class	Subtype	Code	Delivery route	Status	Primary purpose
**IFN alpha/I**	**1b**	NCT04320238	nasal drops	Recruiting	prevention
		NCT04293887	nebulization	Not yet recruiting	therapeutic
	**2b**	NCT04273763	intranasal spray	Active, not recruiting	therapeutic
		NCT04349410	nebulization	Completed	therapeutic
		RPCEC00000308-Sp	nasal drops	Completed	prevention
		RPCEC00000337-En	nasal drops	Completed	prevention
		RPCEC00000318-Sp	systemic	Completed	therapeutic
		NCT04480138	systemic (pegylated)	Recruiting	therapeutic
		NCT04254874	atomization(pegylated)	Recruiting	therapeutic
		NCT04379518	systemic	Recruiting	therapeutic (cancer patients)
	**2b+IFN gamma**	RPCEC00000307-En	systemic	Completed	therapeutic
	**us**	NCT04664010	systemic	Active, not recruiting	therapeutic
		NCT04275388	nebulization	Not yet recruiting	therapeutic
		NCT04534725	intranasal spray	Recruiting	therapeutic
		NCT04251871	aerosol inhalation	Recruiting	therapeutic
**IFN beta/I**	**1a**	NCT04647669	systemic	Not yet recruiting	therapeutic
		NCT04315948	systemic	Active, not recruiting	therapeutic
		NCT04492475	systemic	Completed	therapeutic
		NCT04350671	systemic	Enrolling by invitation	therapeutic
		NCT04521400	systemic	Not yet recruiting	therapeutic
		NCT04460547	systemic	Not yet recruiting	therapeutic
		NCT04449380	systemic	Recruiting	therapeutic
		NCT04330690	systemic	Recruiting	therapeutic
		NCT04552379	systemic (pegylated)	Recruiting	therapeutic
		NCT04732949	nebulization	Recruiting	therapeutic
		NCT04350684	systemic	Enrolling by invitation	therapeutic
		NCT04385095	nebulization	Recruiting	therapeutic
	**1b**	NCT04350281	systemic	Completed	therapeutic
		NCT04276688	systemic	Completed	therapeutic
		NCT04465695	systemic	Recruiting	therapeutic
		NCT04647695	systemic	Recruiting	therapeutic
		NCT04494399	systemic	Recruiting	therapeutic
		NCT04611243	systemic	Recruiting	therapeutic
		NCT04356495	nebulization	Recruiting	therapeutic
		NCT04469491	nebulization	Suspended	therapeutic
	**1a and 1b**	NCT04343768	systemic	Complete	therapeutic
	**us**	NCT04324463	systemic	Recruiting	therapeutic
**IFN lambda/III**	**1a**	NCT04344600	systemic (pegylated))	Recruiting	prevention and therapeutic
		NCT04388709	systemic (pegylated)	Withdrawn	therapeutic
		NCT04354259	systemic (pegylated)	Recruiting	therapeutic
	**us**	NCT04343976	systemic (pegylated)	Enrolling by invitation	therapeutic
		NCT04534673	systemic (pegylated)	Recruiting	therapeutic

us, not specified.

The therapeutic Cuban protocol has included IFN-α (IFN alpha 2b, Heberon ALFA R, Cuba) therapy even though it is currently evaluated in clinical trials for COVID-19 patients (see at https://rpcec.sld.cu/). The first results have been reported by Pereda et al. (101), and although it was not a randomized clinical trial rather a retrospective analysis with a small control group of patients with no IFNα treatment – the major outcomes were a reduction in time needed to obtain a negative qRT-PCR and more satisfactory recovery of treated patients. The abovementioned pieces of evidences suggest that type I IFN can be used both as, therapeutic as well as prophylactic agents against SARS-CoV-2. Its prophylactic effect has a meaningful efficacy as a protective treatment directed to personnel at risk such as the medical community and other vulnerable groups.

Regarding the complexity of type I IFN system, the biology that underlies each specific context, and the rationale for its use in both prophylactic and therapeutic approaches; the ideas conceptualized by Stetson and Medzhitov (12) are truly revealing and enlightening:

-In uninfected cells, type I IFN signaling will activate the “antiviral state” through ISGs expression, which turns the cells more sensitive to the detection and elimination of the potential incoming virus. This may mimic the cellular scenario of a type I IFN prophylactic intervention.

-Once the cell is infected by the virus, type I IFN signals will be integrated with the cell-autonomous detection of viral nucleic acids. These events will allow the intrinsic apoptosis activation pathways to proceed and simultaneously to express ligands that instruct NK cells and cytotoxic lymphocytes to distinguish infected cells from their uninfected neighbors. If the infected cell is unable to commit suicide, there is an alternative path to NK and cytotoxic T lymphocyte in which this cell will be targeted and killed.

Such a context would reproduce the first days after a viral infection wherein type I IFNs could be exogenously administered.

-When lymphocytes themselves are infected, cell-intrinsic viral detection activates apoptosis. In this scenario, coincidence with type I IFNs exogenously delivered would produce a calamitous outcome. Lymphopenia is one of the major deleterious effects of viruses such as SARS-CoV-2 during the late phase of the disease when the infection becomes more systemic, affecting various organs, and with clear evidence of inflammation development (102). Most experts in the art advocate the accurate timing for IFNs therapeutic intervention during viral infections (81, 85, 94, 103, 104). Accordingly, the SARS-CoV-2 infection outbreak has illustrated how dismal turns to be type I IFNs late intervention in terms of poor outcomes (20, 82, 100, 103).

Theoretical contributions of several authors (3, 86, 105–108) have suggested that the role of IFN in the context of COVID-19 is even broader than its contribution as an antiviral and immunomodulatory agent. The ideas described below support this assertion.

## Relationship Between type I IFN and ACE2

The recent discovery that angiotensin-converting enzyme 2 (ACE2) is an ISG (108) has expanded the therapeutic understanding of IFNs role in the COVID-19 treatment because ACE2 is essential for pulmonary and systemic homeostasis in health and in disease conditions (109).

### ACE2 as a Major Component of the Renin–Angiotensin System

The renin–angiotensin system (RAS) is a signaling pathway involved in the regulation of vascular function, including the regulation of blood pressure, natriuresis, and blood volume control (110). RAS is also responsible for local tissue homeostasis by anti-inflammatory, anti-coagulant, anti-proliferation, anti-fibrosis, anti-oxidative stress activities, and anti-apoptosis of epithelial cells (109).

**Figure 4:**
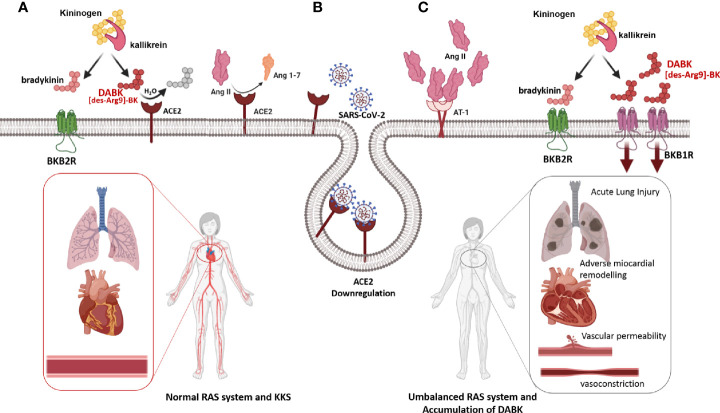
Depletion of ACE2 after SARS-CoV-2 infection **(A)** Under physiological conditions, the RAS allows the harmonic functioning of the cardiorespiratory system, and even the peripheral vascular tone. Under these conditions, the relationship between RAS and Kinin Kallicrein System (KKS) consists of that ACE2 hydrolyzes and inactivates the most active metabolite of bradykinin, DABK, and prevents its signaling through the BKB1R receptor. **(B)** The infection with SARS-CoV-2 produces ACE2 depletion because the SARS-CoV-2-ACE2 complex is internalized into the cytoplasm. **(C)** Depletion of ACE2 produces an increase in its substrates, Ang II, and DABK. The concerted action of Ang II and DABK through signaling induced by their cognate receptors, AT_1_R and BKB1R, respectively, produces an inflammatory state, together with vascular permeability, vasoconstriction, recruitment of inflammatory cells, and pulmonary and systemic damage (125, 126).

The angiotensin-converting enzyme 1 (ACE1) and the homolog ACE2 are two antagonist enzymes of the RAS. ACE1 converts angiotensin I (Ang I) to angiotensin II (Ang II). Both in health and in disease conditions ACE2 receptor and its signaling pathway are an important counter regulatory mechanism of RAS, whose ACE1/Ang II/Ang II type 1 receptors (AT1R) axis mediates vasoconstriction/proliferative status and ACE2/Ang 1-7/Mas axis counterbalance the former by its vasodilator/antiproliferative effects (109) (**Figure 4**).

Ang II is a peptide associated with vasoconstriction, inflammation, fibrosis, and proliferation (109). Its pro-inflammatory effects are mediated through AT1R (110) which transduces a signaling cascade resulting in inflammation (111, 112), vasoconstriction (113), insulin resistance (114) and thrombosis (115–117). Ang II favors the infiltration of macrophages and lymphocytes and also mediates an inflammatory status characterized by increased interleukin (IL)-2, IL-6, tumor necrosis factor-α (TNF-α), IL-1β, IL-18 and overexpression of the NLR family pyrin domain containing 3 (NLRP3) inflammasome (118, 119).

ACE2 is a monocarboxypeptidase that converts Ang II into the heptapeptide Ang 1–7, which by its vasodilator actions on the Mas receptor, opposes the vasoconstriction effects of Ang II and exerts organ protection (109, 120). ACE2 system is a critical protective pathway against heart failure, myocardial infarction, hypertension, systemic and pulmonary hypertension, and cardiovascular complications of diabetes mellitus (3, 121).

### ACE2 Depletion Due to SARS-CoV-2 entrance into the cells

Remarkably, ACE2 is also the entrance receptor of SARS-CoV and SARS-CoV-2 into the cells (122, 123). SARS-CoV-2 undergoes endocytosis with ACE2 as a receptor-ligand complex. ACE2 not only disappears from the cell surface but it also elicits disintegrin and metalloproteinase 17 (ADAM-17) activities, which produce membrane shedding of ACE2 (110). In this context Ang II does not undergo its normal catabolism into Ang 1-7, which means that Ang II is accumulated over its physiological level (117). SARS-CoV-2-mediated down-regulation of ACE2 not only increases Ang II stimulation and contributes to the deleterious hyper-inflammatory reaction of COVID-19 (124), but also increases levels of des-Arg (9)-bradykinin (DABK), which is an active metabolite of bradykinin (BK). DABK is associated with lung injury and inflammation due to its accumulation in the extracellular environment of infected and neighboring cells, where it perpetuates a vicious positive feedback loop of inflammation and injury leading to BK-mediated inflammation and injury (125, 126) (**Figure 4**).

Being ACE2 the front door of SARS-CoV and SARS-CoV-2 (122, 123), it is at the crossroad between SARS-CoV-2 infection and COVID-19 pathogenesis, because in its double role as negative regulator of the RAS homeostasis, and as SARS-CoV-2 receptor, it plays a determinant function in the clinical evolution of COVID-19 patients. Accordingly, the potential capability of type I IFN to induce ACE2 (108), represents an extraordinary opportunity to restore the expression and function of ACE2 and thus, to contribute to RAS homeostasis.

### ACE2 Depletion Elicits Serious Pathological Events in Severe COVID-19 Cases

Dysregulation of RAS is particularly notable in COVID-19 patients with comorbidities wherein in addition to respiratory involvement, multiorgan dysfunction may occur in response to SARS-CoV-2 infection (110). Comorbidities, such as high blood pressure or diabetes, have previously undermined the RAS system (127–129), so that the concomitance with SARS-CoV-2 gives rise to a much worse clinical picture of these patients (130). Disrupted balance of RAS in the COVID-19 context implies that the excess of Ang II, secondary to decreased ACE2 levels, causes pulmonary vasoconstriction, inflammation, cytokine-induced organ damage (131), increased membrane permeability (132), and epithelial cell apoptosis (133). Proinflammatory cytokines together with increased vascular permeability caused by over-activation of the AT1R in the lungs induce acute lung injury, acute respiratory distress syndrome, and could lead to death (117, 134). To support this idea, severe cases of COVID-19 have been reported to have significantly higher systolic pressure as compared to non-severe cases (135), and markedly elevated circulating Ang II levels, linearly correlated with viral load (3, 128, 136) and lung injury (137). These evidences provide a direct association between tissue ACE2 downregulation with systemic RAS imbalance, and the subsequent development of multiorgan damage in SARS-CoV-2 infection (3).

The cytoprotective role of ACE2 is indeed evident in an infectious context, where pre-existing and persistent deficiency of active ACE2 leads to excessive neutrophil accumulation in the lungs, resulting in a hyperinflammatory response and lung damage (107). The cytoprotective function of ACE2 is such that one of the best cytokine storm-driven inflammation animal models is the ACE2^-^/^-^ deficient mouse (138) and that also the loss of ACE2 expression in mutant mice results in enhanced vascular permeability, increased lung edema, neutrophil accumulation, and worsened lung function (139). Accordingly, ACE2 depletion in the cardiovascular system has a negative impact on COVID-19 patients and it is supported by the fact that severe cases of COVID-19 have significantly higher systolic pressure compared to the non-severe cases (135).

The dichotomy of IFN in ACE2 expression and ACE2 internalization pave the way for controversy between two solid criteria, each mutually excluding. On one hand, the relationship between ACE2 and IFN is deleterious as IFN induces ACE2 and thus facilitates the virus entry into the cells. It speculatively means that IFN treatment even during early phases of viremia could reinforce the severity of COVID-19. However, the hypothesis that states that ACE2 upregulation may increase the susceptibility to SARS-CoV-2 entry and may favor a more severe clinical course of the illness through a larger viral burden into the cells remains to be proved (124).

On the other hand, ACE2 induced by IFN contributes to the homeostasis of the RAS system and thus precludes all the consequences of ACE2 downregulation with the resulting increases in Ang II concentration, which elicits a vicious cycle caused by RAS disruption. The inclusion of IFN-α in the treatment protocol in Cuba could explain a low rate of patients complicated at serious stages of the disease (about 7% during the latest outbreak of COVID-19), as evidenced by public data (covid19cubadata.github.io/#cuba) and also, as compared to the 12% of SARS-CoV-2 positive patients who required ICU admission (140). It is noteworthy that this percentage includes a number of patients who did not attend early and therefore did not receive IFN treatment.

Considering ACE2 as an ISG in human epithelial cells (108), the exogenous delivery of IFN could succeed in restoring the ACE2 expression and the homeostasis of the RAS, precluding all the consequences of ACE2 downregulation, which is advantageous regardless of ACE2 being a cellular access point for SARS-CoV-2. ACE2 induced by IFN would contribute to the homeostasis of the RAS system and thus preclude all the consequences of ACE2 downregulation of with resultant increases in Ang II concentration, which elicits a vicious cycle caused by the disruption of the RAS. Experimentally, it has been demonstrated in rats that a 3-day treatment with IFN-α led to a permanent and statistically significant decrease in blood pressure and heart rate reduction (141).

Retrospective studies of COVID-19 patients treated with type I IFNs could help document the evidence of IFN effect in patients with and without comorbidities, specifically regarding easy-to-record clinical and laboratory parameters, all of which would support the wide range of IFNs effects beyond their antiviral and immunomodulatory roles. The shreds of evidence concerning the extensive therapeutic use of IFN-α in Cuba, as part of the national treatment guideline during the COVID-19 pandemic, demonstrate a significant reduction in the number of patients progressing to severe forms of COVID-19 (101).

## The Relationship Among Type I IFN, Neutrophil Extracellular Traps, and IL-17

**Figure 5:**
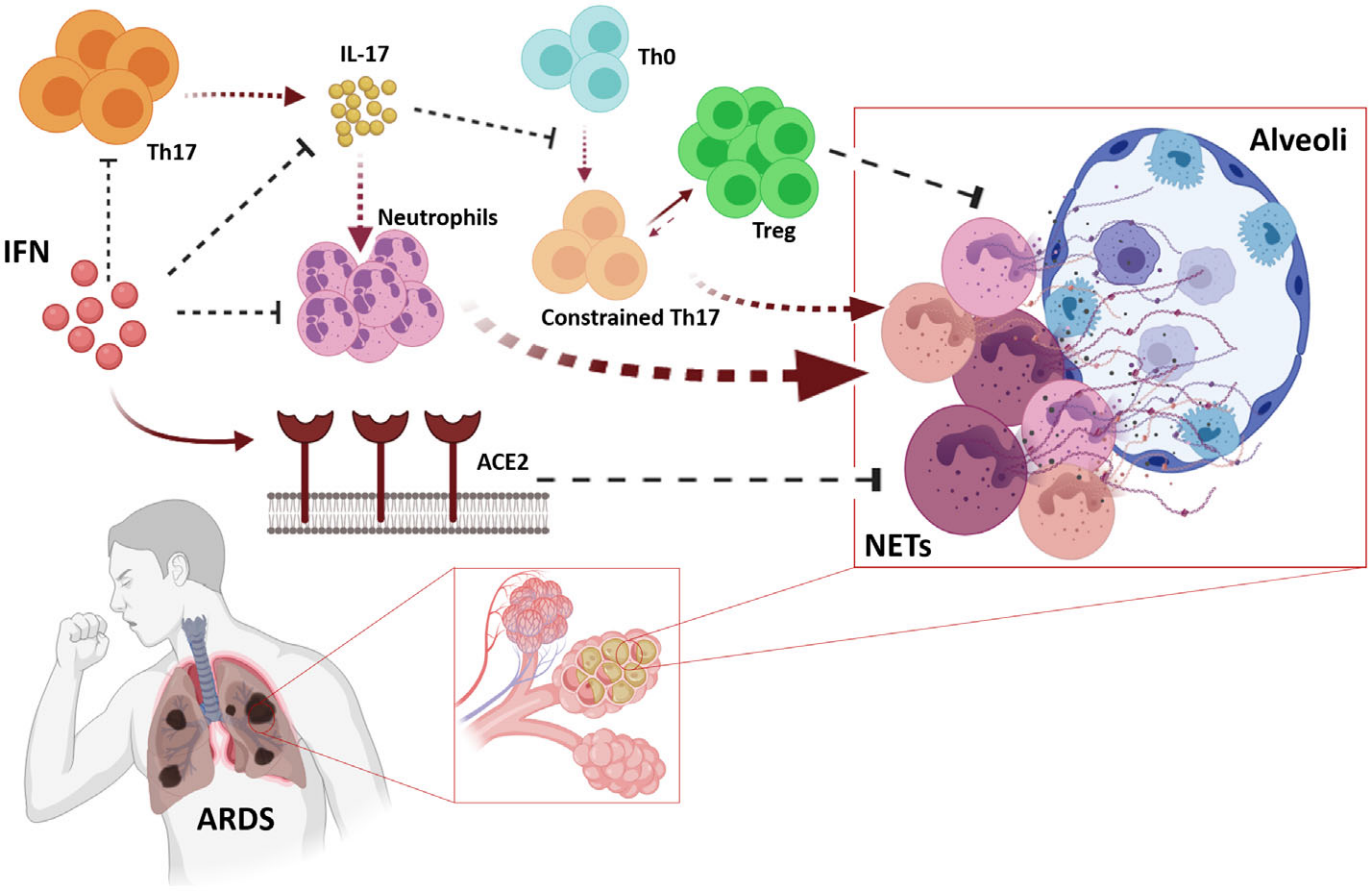
Pleiotropic effects of IFNs may cope with Acute Respiratory Distress Syndrome (ARDS). IFNs impair biological responsiveness of neutrophils, and suppress IL-17 expression which also avoids maladaptive neutrophil recruitment affecting neutrophil-mediated inflammation regulated by T cells expressing IL-17. The relative scarcity of IL-17 avoids clonal expansion of Th17 cells, which allows Treg to perform its counterbalancing role on immune homeostasis. IFN-induced ACE2 contributes avoiding the pathogenic effects of neutrophils in lung parenchymal.

Neutrophil extracellular traps (NETs) cause the most severe cases of COVID-19, even in pediatric cases (142–144), which is reinforced by the fact that high neutrophil to lymphocyte ratios are strongly associated with SARS-CoV-2 pathogenesis (145). As IFN-α alters the biological responsiveness of neutrophils (146), it could indirectly contribute to the major salutary effects of this treatment. Additionally, it has been demonstrated that IFN-α potently suppressed IL-17 production and this impairs naive T cells differentiation onto T-helper 17 (Th17) cells (16, 147, 148). Thus the arm of neutrophil-mediated inflammation regulated by T cells secreting IL-17 is another robust target of IFN in the context of COVID-19 pathogenesis. In addition to this, the effect of reduction of IL-17 encompasses Th17 cells, which remains constrained, thus facilitating the role of regulatory T cells in counterbalancing the homeostasis of the immune system (105). Increased IL-17 levels and other Th17 cell-related pro-inflammatory cytokines have been reported in patients with SARS-CoV-2 as one of the main components of the cytokine storm, associated with high viremia and disease severity (106, 149). Therefore, the indirect dual effect of IFN by blocking IL-17 expression (86) and accordingly, reducing neutrophilia and Th17 cells differentiation has been advocated as a rational therapeutic element in the treatment of COVID-19 (106) (**Figure 5**).

Dynamic variation of pulmonary ACE2 is also required to control the neutrophilic inflammation of the host in response to infection (107), so the suitable timing intervention with IFN could contribute to restoring ACE2 levels and thereby contribute to control neutrophil infiltration.

## Future Perspectives

Considering ACE2 is an ISG, one of the most relevant clinical evidences of IFN therapy would be its effects on cardiorespiratory parameters, such as oxygen saturation and blood pressure. These data may ultimately assure the real contribution of IFN-induced ACE2 in the context of SARS-CoV-2 infection, which undermines the RAS (3, 150). Furthermore, as the ACE2 gene lays on chromosome X (109), as well as another protein involved in the induction of IFN expression, NEMO (Ikbkg) (151), it would be interesting to assess the inter-gender response to IFN therapy. Irrespective to the rationale and the potential therapeutic impact of IFNs during COVID-19, it is important to know that some individuals are carriers of mutations in the IFN signaling system proteins as well as there are some individuals with autoantibodies against interferons type I, in which the most severe forms of the disease have been found (151, 152). Although they are not very frequent, these peculiarities would prevent an effective therapeutic response to treatment with interferons.

### Take Home Messages

- During a viral infection, the most prominent cytokines produced are IFNs, thus, as SARS-CoV2 impairs IFNs endogenous production, the exogenous delivery of recombinant IFN is a rational approach. IFN primed cells may abolish the SARS-CoV-2-induced block in innate immune activation.

-The dual role of IFNs -direct inhibition of viral replication and eliciting an immune response to clear virus infection is accompanied by other important effects: Type I IFNs induce ACE2, blocking IL-17 signaling, and also impair biological properties of neutrophils. All the abovementioned properties and/or effects are strongly associated with SARS-CoV-2 and COVID-19 pathophysiology.

-Desensitization role of IFN: The plethora of actions mediated by IFNs include desensitization of immune response to avoid collateral damage. This fact is not only an important evidence of the accuracy required for IFN-based therapeutic approaches but also an opportunity to foster the concerted actions with other immunomodulatory interventions contributing to the restoration of the immune system homeostasis.

### Other Bounties of IFN in the Context of COVID-19

As a central link between the innate and adaptive immune systems, IFNs are mandatory for regulating the activation and functions of various immune cell populations (16). Taking into account that IFNs are promoters of the survival and effector functions of T cells, the impaired T cell responses- as lymphopenia- during COVID-19, may obey to an impaired IFN production. Bearing in mind the role of IFNs in the development of regulatory T cells, and the inverse correlation between regulatory T cell count and the disease severity in COVID-19 patients (105), it may be reasonable to consider IFN dysregulation as an underlined event in COVID-19 pathogenesis which deserves a replacement therapy based on IFNs.

## Conclusions

Early therapeutic, and even prophylactic, IFN interventions during COVID-19 could reduce disease severity and contribute to viral clearance, in turn avoiding multi-organ damage and patient death. IFN therapeutic administration during SARS-CoV-2 infection not only accounts for the antiviral and immunomodulatory effects of this drug, but it is also an opportunity to restore the SARS-CoV-2-impaired IFN signaling system, and thereby to promote the occurrence of other ISGs-mediated mechanisms that are relevant in the context of COVID-19. It also means that the antiviral and immunomodulatory effects of IFNs synchronized with other IFNs’s benefits for the direct or indirect control of inflammatory cytokines, neutrophilia, regulatory T cells, and the induction of ACE2 expression, may help to mimic a physiological antiviral response, with an intact IFN signaling system.

## Author Contributions

DG-d-B and DR-A contributed to the conceptualization of the subject, literature search, graphical designs of figures, and writing the manuscript. FDM-B contributed to the critical review of clinical and epidemiological data. JB-A and GG-N contributed to the conceptualization of the subject and reviewed critically the manuscript. All authors contributed to the article and approved the submitted version.

## Conflict of Interest

Author GG-N is a Topic Editor of this Research Topic.

The remaining authors declare that the research was conducted in the absence of any commercial or financial relationships that could be construed as a potential conflict of interest.

## In Memoriam

The authors want to pay tribute to the memory of their professor and friend, Dr. Pedro Lopez-Saura, who was a pioneer and prominent clinical investigator of interferon research in Cuba.
